# In and Al Schottky Contacts Comparison on P-Type Chlorine-Doped CdTe

**DOI:** 10.3390/s21082783

**Published:** 2021-04-15

**Authors:** Igor Vasylchenko, Roman Grill, Marián Betušiak, Eduard Belas, Petr Praus, Pavel Moravec, Pavel Höschl

**Affiliations:** Institute of Physics, Faculty of Mathematics and Physics, Charles University, Ke Karlovu 5, CZ-12116 Prague 2, Czech Republic; grill@karlov.mff.cuni.cz (R.G.); majo.betusiak@gmail.com (M.B.); eduard.belas@mff.cuni.cz (E.B.); praus@karlov.mff.cuni.cz (P.P.); moravec@karlov.mff.cuni.cz (P.M.); hoschl@karlov.mff.cuni.cz (P.H.)

**Keywords:** CdTe, transient current, space charge, polarization

## Abstract

The performance of the CdTe radiation detectors heavily relies on the method of contact preparation. A convenient research method addressing this problem is the laser-induced transient current technique. In this paper, we compare the performance of two CdTe crystals which underwent different metallization processes. We showed that appropriately designed Au/Al contacts induce much less bulk polarization than commercial Pt/In electrodes under the same working conditions and can thus provide a convenient alternative to the industry standard. The comparison was based on the monitoring of the time-dependent sensor polarization measuring transient currents excited by above-bandgap laser illumination complemented by the Am 241 gamma spectroscopy. The theoretical analysis of current waveforms and radiation spectra enabled us to determine the charge carrier mobility, mobility-lifetime products of electrons and holes, and temporal and bias dependence of the space charge formation.

## 1. Introduction

Modern gamma- and X-ray semiconductor radiation detectors are manufactured from different materials [1]. Among them, CdTe belongs to the most prosperous materials with excellent detection properties [2,3]: high mobility-lifetime product and high signal-to-noise ratio. When attaining a high resistivity by doping [4], the group-VII dopants, especially chlorine, are more favorable than the group-III ones [5] due to their better stability and propensity to form p-type material [6]. The p-type CdTe has an advantage over the n-type manifesting in an improved charge collection efficiency of holes often degraded by hole trapping on a hole trap positioned near the middle of the bandgap [7].

In spite of the undoubted progress in CdTe radiation detector technology, there are remaining issues that need to be addressed in this type of material. One of them is the polarization of biased detectors equipped with Schottky contacts, which results in signal deterioration in time [8,9,10]. The polarization is caused by the space charge formation in the depleted detector inducing the screening of the applied bias and leading to an appearance of an inactive region (dead layer) under one of the contacts. Another issue is the need to suppress the leakage current and thermal noise in the detector. An alternative approach depositing quasi-ohmic contacts results in the fast onset of the leakage current at only tens of volts biasing [11]. In spite of relatively high work function of the CdTe (5.4–5.7 eV), such contacts can be made by the electroless deposition of Au or Pt films. The latter are known to form a Cd-depleted region near the surface of the semiconductor [12,13], which resembles a heavily doped p-type interlayer and thus improves ohmicity. Lastly, the exact contact properties such as their mechanical and electrical stability rely heavily on the method of metal deposition as well as the surface preparation [13].

Convenient methods to study the charge dynamics including the polarization phenomena in biased detectors are the transient charge technique (TChT) [14], transient current technique (TCT) [15], and laser-induced transient current technique (L-TCT) [16,17,18,19]. Among these, the L-TCT excited with the above-bandgap laser pulses has several advantages. Namely, it provides an appealing signal-to-noise ratio due to the synchronization between the laser pulse and the measurement triggering. The detailed study of current transients makes the determination of the lifetime and mobility of excited free carriers, the space charge and electric field profiles possible. The excitation at a well-defined time delay after the switch-on bias can be used for the determination of the temporal evolution of these quantities [16].

The primary aim of this paper is the optimization of the CdTe detector with respect to the abovementioned disadvantages—polarization of detectors with Schottky contacts and high leakage current of the detectors with quasi-ohmic contacts. Based on this premise, we investigate two p-CdTe radiation detectors equipped with different electric contacts metallization—commercially available (In, Schottky barrier 1.38 eV [20]) and in-house (Al, 0.973 eV [10]) Schottky contacts. We show that the polarization can be significantly suppressed by substituting In by Al and proper contact manufacture while at the same time the leakage current remains acceptable for up to 400 V of detector biasing. We simultaneously investigate the space charge formation by L-TCT in both sensors and present an improved method combining L-TCT and Am−241 gamma spectra analysis, which is convenient for the characterization of polarizing detectors.

**Figure 1:**
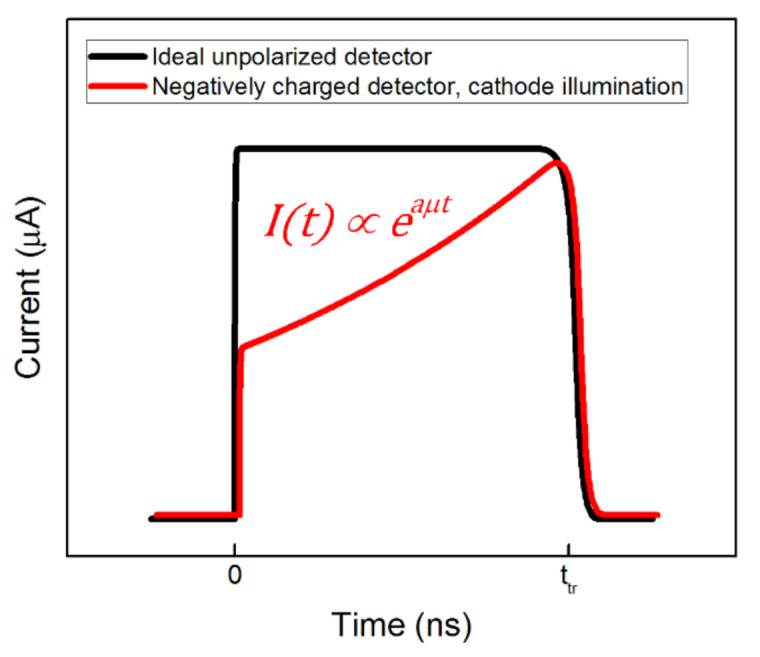
Example of electron current waveforms (CWFs) in an ideal nonpolarized (black) and negatively charged polarized detector (red). The descending part of the CWF is smeared by the diffusion. The *t_tr_* is set to the point of inflexion. Typically, the current maximum reaches several μA and *t_tr_* is in the range of tens to hundreds of ns.

Current waveforms (CWFs) measured by L-TCT have different shapes depending on the electric field profile. Examples of CWFs are shown in Figure 1. Assuming an ideal unpolarized planar detector and very long carrier lifetime *τ* significantly exceeding the time of the passage of the charge through the detector, transit time *t_tr_*, the current is nearly constant over the time until the charge reaches the opposite electrode at *t_tr_* and drops to zero. In this case, *t_tr_* is defined as:

(1) $$t_{tr}=\frac{L^{2}}{\mu U}$$

where *L* is the detector thickness, *μ*—charge carrier mobility, and *U*—applied bias. In a homogeneously charged planar detector, an exponential CWF arises. In particular, if the detector is negatively charged and electron current transients are measured, the CWF attains an ascending profile and the transient current time dependence can be expressed as [21]:

(2) $$I\left(t\right)\propto e^{\left(a-\frac{1}{\mu \tau }\right)\mu t},$$

where *a* represents the slope of the electric field along the detector thickness, defined as:

(3) $$a=\frac{eN}{\varepsilon_{0}\varepsilon_{r}}$$

where *e* is an elementary charge, *N*—negative space charge density (expressed in cm^−3^), *ε_0_*—vacuum permittivity, and *ε_r_*—relative permittivity. Simultaneously, the transit time *t_tr_* transforms to a more complex form [21]:

(4) $$t_{tr}=\frac{1}{a\mu }\ln\left(\frac{1+\frac{aL^{2}}{2U}}{1-\frac{aL^{2}}{2U}}\right)$$

The charge collection efficiency (CCE) is derived from the Gamma spectra using the common formula considering both electron and hole contributions and an inhomogeneous electric field *E*(*x*) that is distorted due to the polarization. The spatially dependent charge collection efficiency *η*(*x*) of a charge created by the interband photoexcitation at the depth *x* in the planar detector can be calculated through the expression [22,23]:

(5) $$\eta \left(x\right)=\frac{1}{L}\left\{\int_{x}^{L}exp\left[-\int_{x}^{x_{e}}\frac{d\xi }{\mu_{e}\tau_{e}E\left(\xi \right)}\right]dx_{e}+\int_{0}^{x}exp\left[-\int_{x_{h}}^{x}\frac{d\xi }{\mu_{h}\tau_{h}E\left(\xi \right)}\right]dx_{h}\right\}$$

Cathode (anode) is at *x* = 0 (*x* = *L*). To avoid causal confusion, we used positive *U* and *E* values overall in the paper. Equation (5) represents a generalized *η*(*x*) to the formula derived by Hecht [24] where the homogeneous electric field *E* = *U/L* was used.

The full charge collection efficiency (CCE) determined at the radiation spectroscopy is calculated by the integration:

(6) $$CCE=\frac{\alpha }{1-e^{-\alpha L}}\int_{0}^{L}e^{-\alpha x}\eta \left(x\right)dx$$

where *α* is the attenuation coefficient of the radiation illuminating the cathode side. Combining Equations (5) and (6), the CCE was fitted to the experimental data, optimizing mobility-lifetime products *μ_e_τ_e_* and *μ_h_τ_h_*, and *E(x)*.

Obvious models used at the simulation of the charge drift in polarizing radiation detectors base the analysis on a homogeneous space charge formed in the depleted region and uncharged inactive layer when the electric field is screened within the detector thickness [21,25,26]. We follow these models in our analysis. In addition, with the aim to draw *E(x)* closer to real electric field profiles, we improved the model slightly and took into account that the electric field is not fully screened in the inactive region and a residual field lasts out there. The reason for this modification stems from the necessity to transfer the charge supplied by the blocking contact or created by the thermal interband excitation in the depleted layer through the inactive region to the contact. This model improvement is especially important in the CCE analysis at low bias where the characteristic bending of CCE vs. bias was observed. The corresponding formula then acquires a more complex form. Defining the depletion width:

(7) $$W=\sqrt{\frac{2\left(U-E_{r}L\right)}{\left|a\right|}}$$

where *E_r_* is the residual electric field in the inactive region; the electric field distributed within the whole detector is expressed by the formula:

(8) $$E\left(x\right)=\left\{\begin{array}{c} \frac{U}{L}+a\left(x-\frac{L}{2}\right);\;\;\;\;\;\;W>L\\ a\;max\left(x+W-L,0\right)+E_{r};\;W\langle L\;\&\;a\rangle \;0\;\;\\ -a\;max\left(W-x,0\right)+E_{r};\;\;\;W<L\;\&\;a<0 \end{array}\right.$$

The value of parameter *a* > 0 (*a* < 0) represents negative (positive) space charge. The residual electric field *E_r_* is fit via the trial form:

(9) $$E_{r}=\frac{U}{L}\frac{1}{1+\kappa U}$$

which complies with a necessary ohmic character of contacts at the lowest bias and saturates at a large bias. The only parameter *κ* defining the crossover between low and high bias is optimized.

## 2. Materials and Methods

**Figure 2:**
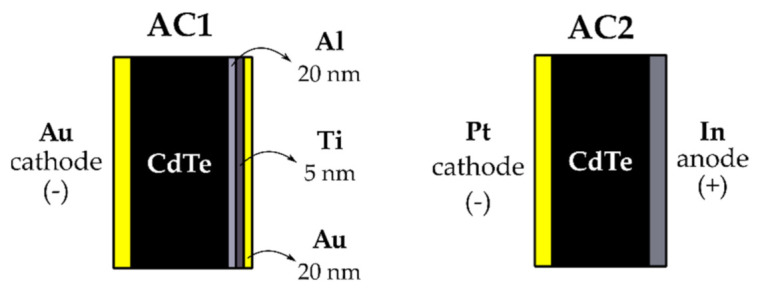
Planar contacts on AC1 (**left**) and AC2 (**right**) samples.

We conducted measurements on commercially available Acrorad p-CdTe crystals. Two types of sensors, further denoted as AC1 and AC2, were investigated. AC1 samples with dimensions of 6 × 6 × 1 mm^3^ were purchased without contacts with marked A and B faces. We deposited semitransparent Au/Al contacts on them using the following procedure. At first, AC1 samples were masked by chemically inert lacquer (photoresist AZ 1350) leaving the A face exposed to the subsequent electroless gold deposition, for which crystals were put into 1% aqueous solution of AuCl_3_ for 1 min. After rinsing in distilled water and dissolving the lacquer in acetone, they were additionally cleaned in fresh acetone and isopropanol and dried. Then, the samples were masked for the second time with the B face being uncovered. Before the contact metal sputtering (Al, Ti, Au), the sample surface was cleaned by He plasma. The resulting structure after the same washing and cleaning, as carried out at Au contact, is depicted in Figure 2. It consists of Al used as a primary contact metal, an adhesive Ti interlayer, and protective/conductive Au covering. Commercial AC2 samples with dimensions 10 × 10 × 1 mm^3^ were received with the planar Pt/In contacts. A method of contact preparation as well as prior surface treatment is not known.

**Figure 3:**
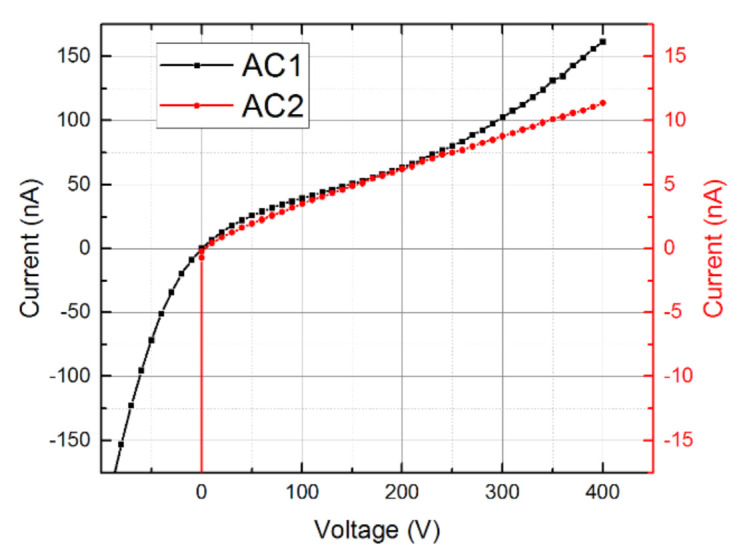
I-V curves of the used samples.

For both samples, we measured I-V curves, as plotted in Figure 3, showing rectifying characteristics ruled by the reverse-biased Schottky contacts on the anode. Respective bias polarities corresponding to the lower leakage currents are denoted in Figure 2. All L-TCT and gamma spectra measurements were performed in the thermally stable media (21 °C ± 1 °C) using these electric configurations.

**Figure 4:**
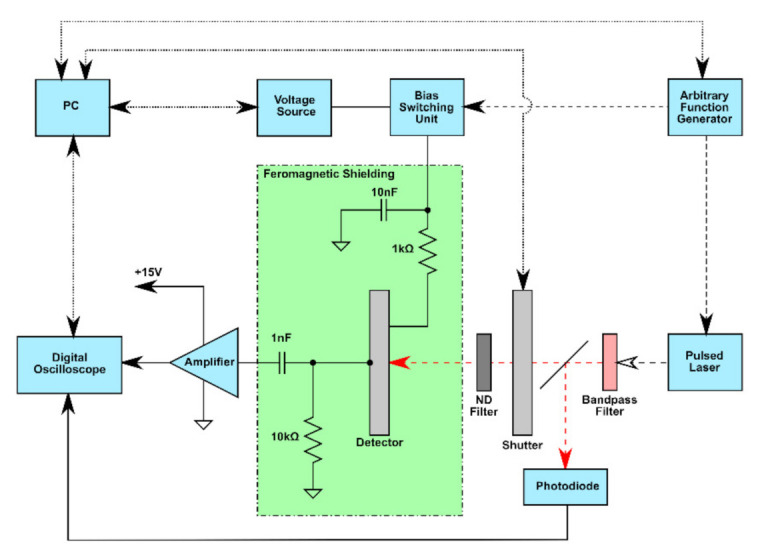
Laser-induced transient current technique (L-TCT) measurement setup.

Transient currents were measured in the L-TCT setup shown in Figure 4. Illumination of the sample’s cathode was performed by the SuperK compact supercontinuum laser (2 ns pulse width, ~30 mm^2^ spot area) combined with optical filter (670 nm). Additionally, neutral density disc filter was placed in the beam line for the intensity attenuation of the laser pulse. In the present work, laser pulses with the output energy of 0.4 nJ and a 100 Hz repetition rate were used to induce all CWFs in the detector and this light intensity did not disturb internal electric field.

Due to the above-bandgap excitation, the light was absorbed in a thin layer (<1 μm) below the interface. Holes were collected immediately at the cathode and only electrons drifted through the biased detector toward the anode, inducing transient current. The current waveforms were recorded by a LeCroy ultrafast digital sampling oscilloscope (40 Gs/s, resolution up to 11 bits, 4 GHz bandwidth) triggered by the optical pulses (photodiode).

**Figure 5:**
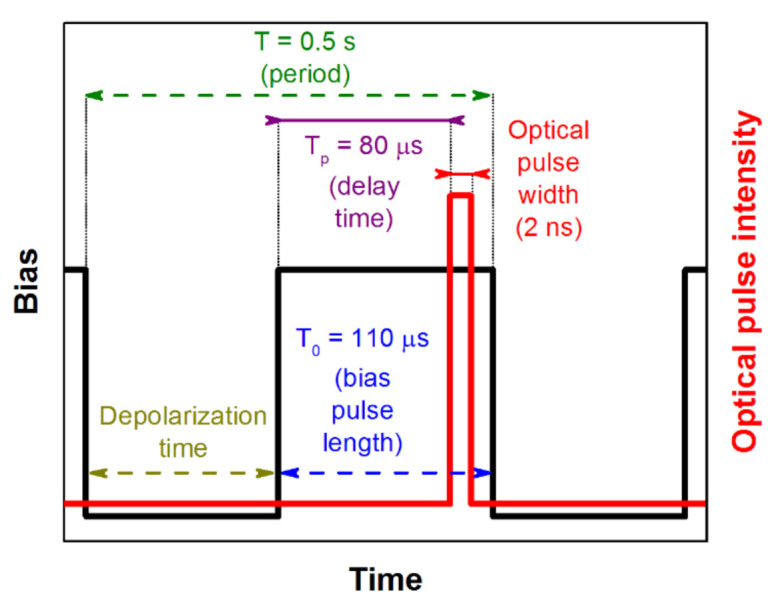
Diagram of the pulsed bias cycle.

We conducted L-TCT measurements in pulsed bias (PB) and direct bias (DB) modes. In the first case, as shown in Figure 5, illumination and the bias switching were carried out in cycles with the period T = 0.5 s. Each cycle starts with the zero bias for the time interval, referred to as the depolarization time. During this interval, the space charge built up in the previous bias pulse dissipates, therefore the next measurement was not distorted by it. After this delay, the bias was switched on for the rest of the cycle using the bias pulse length T_0_ = 110 μs. Finally, at the delay time T_p_ = 80 μs after switching on the bias, the laser pulse was released on the sample and the particular waveform was measured and stored. The delay T_p_ = 80 μs was used as the minimum time sufficient for the stabilization of the applied bias. Waveforms presented in this paper were obtained by averaging over 1000 cycles (500 s), representing the single measurement.

In the DB mode, the constant bias was applied to the sample. CFWs are measured continuously after a given period of biasing T_b_, which is varied in the interval T_b_ = 1–60 min. Data were acquired during a 10 s interval with a 100 Hz (laser pulse) repetition frequency in this regime. Before switching to another bias, zero voltage was applied for at least 2 min to suppress residual polarization.

Gamma spectra were measured using an Am 241 radiation source (89 kBq). The detected count rate was less than 2000 s^−1^. This is significantly below the maximum count rate at which the threshold effect might disturb the detection. The setup for gamma spectroscopy consisted of a Keithley 2410 as a high voltage source and an Ortec 671 shaping amplifier connected to an Ortec multichannel analyzer. Measurements were taken at DB with T_b_ = 1 min and 2 min depolarization at zero bias until the next cycle with another bias started similarly as in DB L-TCT.

## 3. Results

**Figure 6:**
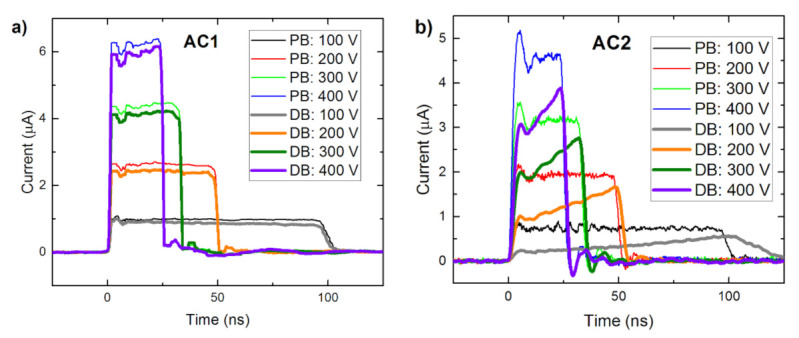
Bias dependence of (**a**) AC1 and (**b**) AC2 waveforms measured in pulsed bias (PB) mode and direct bias (DB) mode after 1 min biasing.

In the first experiment, we measured the bias dependence of CWFs of both AC1 and AC2 detectors biased at 100–400 V in PB (see Figure 5) and DB modes. Figure 6a shows current waveforms measured on sample AC1. We saw that the current waveforms were similar in both modes and no visible polarization phenomena were identified. Simultaneously, the waveforms are nearly flat at the time scale limited by the transit time up to 100 ns, which points to the long electron lifetime and testifies to the high quality of the material. The electron mobility *µ_e_* = 1040 cm^2^/Vs characterizing both AC1 and AC2 samples was evaluated through Equation (1).

In contrast to sample AC1, the waveforms measured on sample AC2, plotted in Figure 6b, deteriorated in the DB mode markedly. In particular, the current rose over time due to the negative space charge formation induced by the blocking Schottky contact at the anode and the inhomogeneous electric field, which is stronger near the anode—i.e., polarization of the detector occurs.

**Figure 7:**
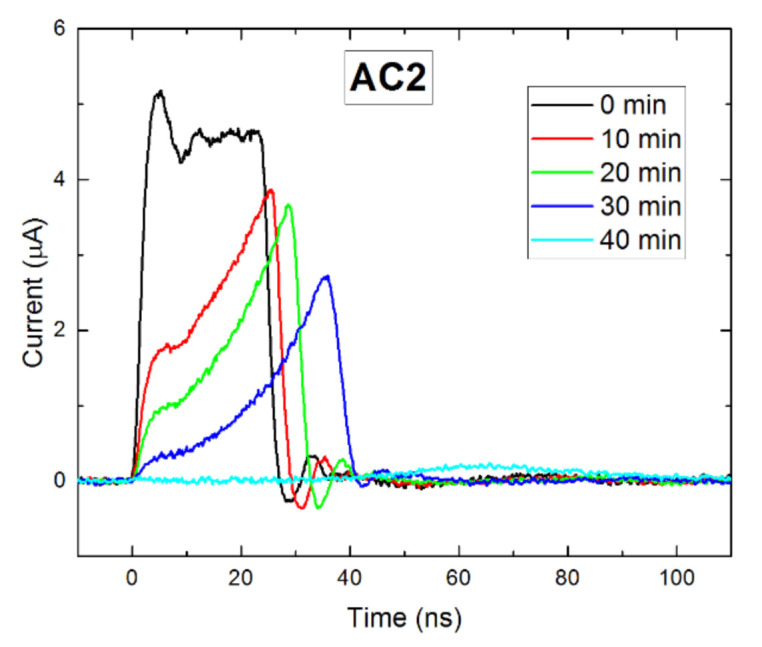
Time evolution of AC2 waveforms at 400 V bias.

In the next experiment, we further investigated the impact of the detector polarization over time. Figure 7 shows the time evolution of the AC2 current waveforms at 400 V bias. It was seen that the signal degraded when the sample became more polarized. After 40 min biasing, the current dropped drastically, due to the appearance of the inactive region near the cathode. Equivalent measurements on sample AC1 showed that the current waveforms remained unchanged for at least 60 min.

**Figure 8:**
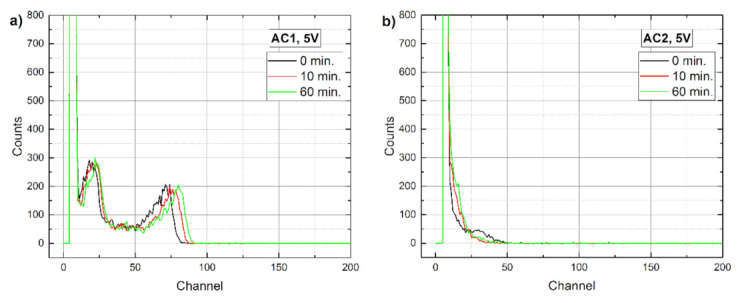
Time evolution of Am 241 spectra measured at 5 V on (**a**) AC1 and (**b**) AC2 samples.

**Figure 9:**
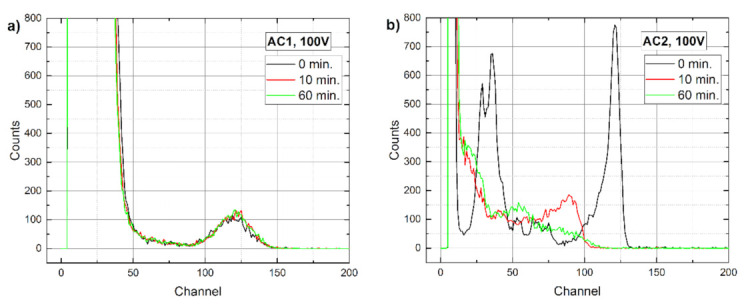
Time evolution of Am 241 spectra measured at 100 V on (**a**) AC1 and (**b**) AC2 samples.

Similar trends were observed also in Am 241 gamma spectra measurements, the results of which are presented in Figure 8 and Figure 9. At the low detector bias shown in Figure 8, the AC1 sample had a much better resolution than AC2, whereas at 100 V (Figure 9) the opposite is true. However, the performance of AC2 degraded with time in both cases while the AC1 spectra remained practically unchanged in the same time span. In our opinion, the reason for the low quality of the AC1 100 V spectrum corresponds to the increasing noise in Au/Al contacts, which must be reduced before the detector could be used at this bias.

## 4. Discussion

**Figure 10:**
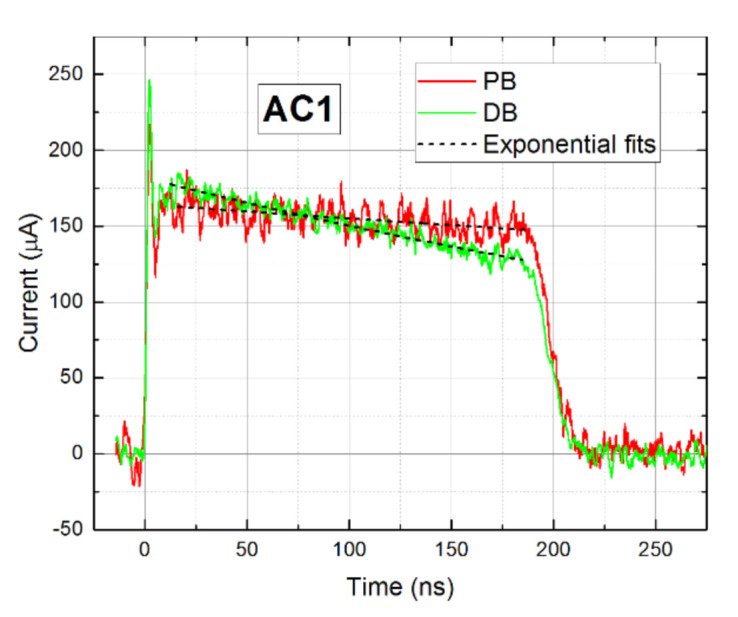
Current waveforms of sample AC1 at 50 V measured at PB and DB after 1 min of biasing. Fits of Current waveforms (CWFs) are shown as well.

With the aim to investigate the detectors in more detail, we performed an extensive theoretical analysis of collected data combining both L-TCT and Am 241 spectroscopy on both samples. At first, we completed the L-TCT measurement on sample AC1 at the low bias where the CWF slope may be visualized more distinctly. We show in Figure 10 the CWFs measured at 50 V at PB, see Figure 5, and DB after 1 min biasing together with exponential fits. Assuming zero space charge in the sample measured at PB and using Equation (2), we evaluated the electron lifetime *τ_e_* = 1.7 μs from the damping of the PB CWF, which yields *µ_e_* = 1040 cm^2^/(V × s) in *μ_e_τ_e_* = 2.0 × 10^−3^ cm^2^/V. The measurement after 1 min shows a clear evolution of the CWFs induced by the space charge. Evaluating the fit according to Equations (2) and (3) and considering the fixed *τ_e_* derived from PB, we obtained the positive space charge density *N* = 7.2 × 10^9^ cm^−3^. A similar *N* was estimated from the CWFs measured at 100 V. We found out that the space charge formed after 1 min biasing did not evolve further and remained stable for at least one hour. We conclude that sample AC1 experiences weak polarization by the positive space charge early after the biasing probably induced by a blocking cathode. The charge remains low and stable both with enhanced bias and extended time. The low charge did not notably disturb the CCE with an operational bias.

Current transients of holes were measured at AC1 by illuminating the anode as well, but the signal depreciated due to a low-frequency noise (not shown in this paper). It is well-known that the TCT measurement of holes is more problematic in CdTe than that of electrons due to the low hole drift mobility and signal-to-noise ratio. Despite the noise, the hole transit time could be determined and the hole mobility *μ_h_* = (76 ± 6) cm^2^/(V × s) was established, which is close to the value *μ_h_* = 80 cm^2^/(V × s) [18] measured by L-TCT on the same material.

**Figure 11:**
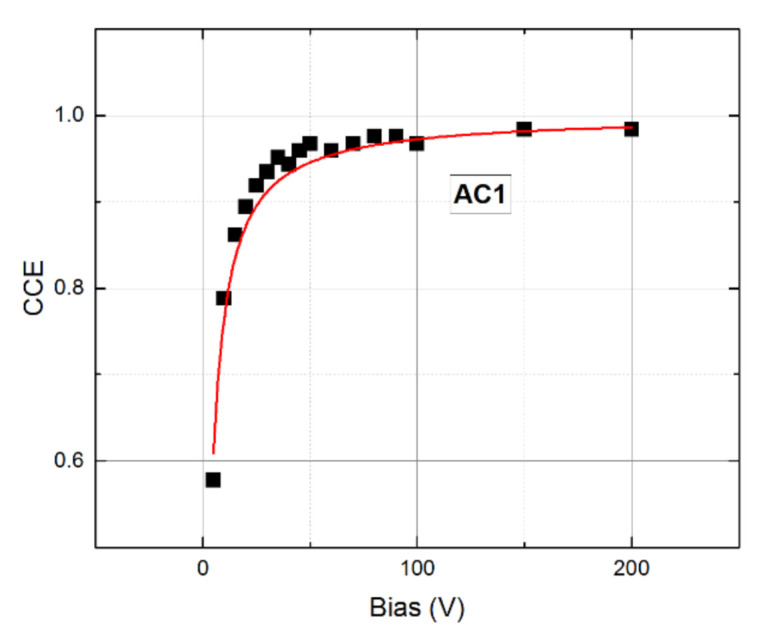
Hecht equation fit of the charge collection efficiency (CCE) on sample AC1.

Characteristics of the sample AC1 evaluated by PB L-TCT, *τ_e_*, and *N* were subsequently implemented in the Hecht equation fit using the charge collection efficiency (CCE) derived from the bias dependence of the gamma spectra (not shown in the paper). Considering the finite attenuation coefficient of Am 241 gamma photons with the energy 59.5 keV, which is estimated to *α* = 38 cm^−1^ in CdTe [3], both electrons and holes were taken into account at the calculation of collected charge in the 1 mm thick sample. The corresponding fit of the CCE of sample AC1 was performed following the theory depicted above, see Equations (5)–(8), in which *μ_e_τ_e_* and *N* specified by L-TCT were fixed. The only optimized parameter thus remained *μ_h_τ_h_*, which resulted in *μ_h_τ_h_* = 6.1 × 10^−4^ cm^2^/V. The respective fit was plotted together with the experimental points in Figure 11. Combining *μ_h_* and *μ_h_τ_h_*, the hole lifetime *τ_h_* = 8 μs was obtained. The measured hole L-TCT signal allowed us to estimate *τ_h_* > 6 μs, confirming the validity of the Hecht equation fit.

In the case of the positive space charge, the inactive region formed near the anode and residual electric field *E_r_* did not markedly affect the CCE. Nevertheless, the positive charging yields in the slight enhancement of CCE at low bias and the involvement of this feature in the theory improves the quality of the fit. Discussion of this effect with examples may be found in [25]. As the Am 241 gamma radiation was absorbed near the cathode, the hole contribution to the total CCE made up 24% and the determined *μ_h_τ_h_* may be burdened by an appreciable error. Since the original materials of both samples are the same, determined mobility-lifetime products were used in the simulations carried out on sample AC2 as well.

**Figure 12:**
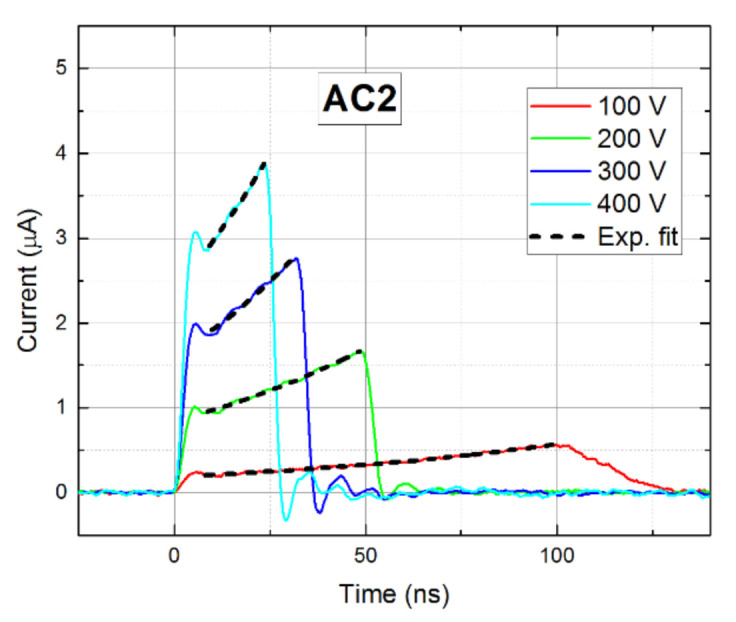
Exponential fits of waveforms measured in DB mode after 1 min biasing.

**Figure 13:**
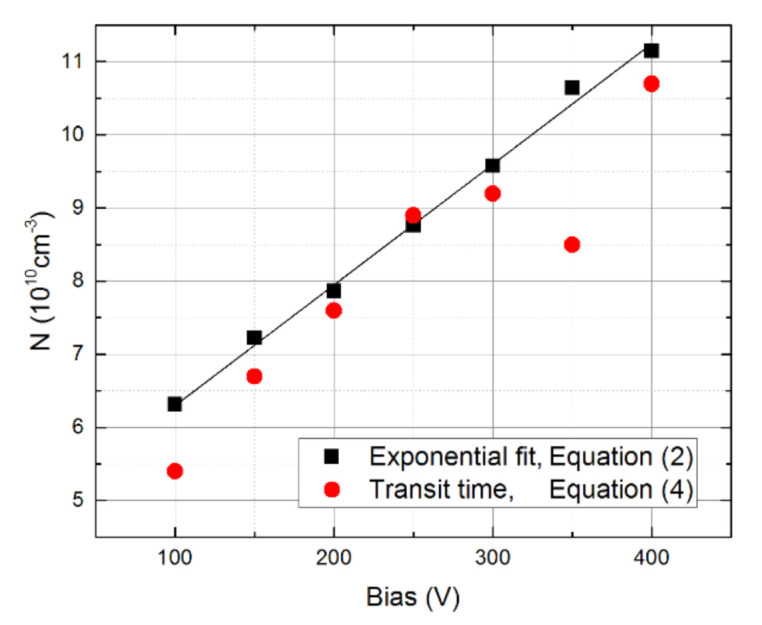
Bias dependence of the normalized space charge density in AC2 derived from the CWF slope (black squares) and from the transit time (red bullets). Linear fit corresponds to the equation *N*(cm^−3^) = 4.62 × 10^10^ + 1.65 × 10^8^ × Bias (V).

Once we fixed transport properties on the nonpolarized sample AC1, we turned our attention to the sample AC2, especially to its polarization. We quantitatively determined the detector polarization using the waveform slope. In contrast to AC1, where weakly descending CWFs point to the positive space charge formation, AC2 reveals significant negative charge polarization. Assuming constant space charge density distribution in the detector’s depleted bulk, the CWF shape was fitted by the theoretically predicted single exponential function—see Equation (2). The respective fits are shown in Figure 12. The growth rates were subsequently used along with Equations (2) and (3) to determine the space charge density *N* at different biases. It can be seen in Figure 13 that, surprisingly, *N(U)* reveals a nearly linear course, which may be fitted by the straight-linear form:

*N*(cm^−3^) = 4.62 × 10^10^ + 1.65 × 10^8^ × *U*(*V*). (10)

The reason for the linear course of *N*(*U*) has not been resolved yet. It will be the subject of future studies.

An independent way to determine the space charge density may be linked to Equation (4), where the polarization parameter *a* may be determined with the transit time. The result of this calculation is plotted with red circles in Figure 13. We can see that this approach shows a significantly larger scatter than the previous treatment, especially at larger biases (>250 V). Nevertheless, the general trend of the space charge evolution was maintained.

**Figure 14:**
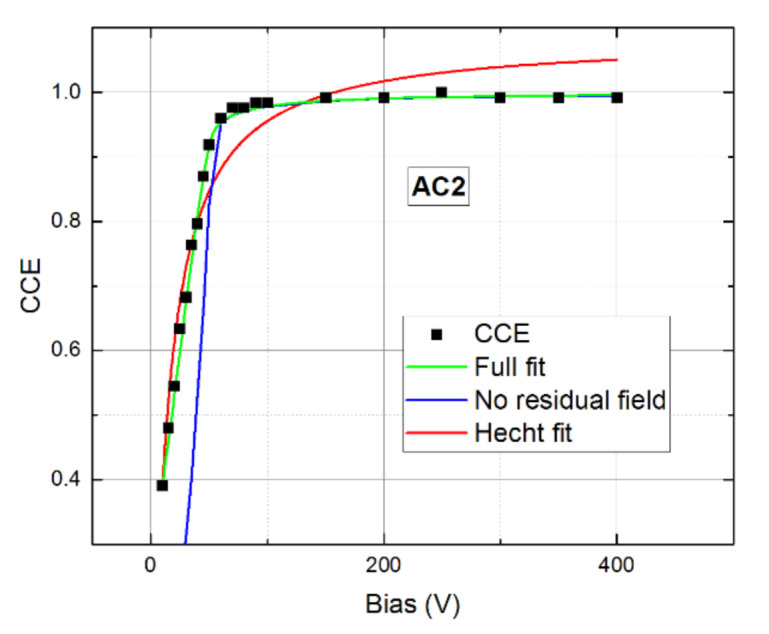
Bias dependence of the charge collection efficiency (CCE) on sample AC2. Full fit is plotted by the green line. The fit with the neglected residual field is blue. The fit with the obvious Hecht equation is red.

All detector properties determined on both AC1 and AC2 samples were used at the fit of CCE of Am 241 gamma radiation. The experimental points were fit with the theoretical forms defined by Equations (5)–(9). There was only one optimized parameter *κ* defining the magnitude of the residual electric field in the inactive region. In addition, the space charge was slightly amended since the appearance of the inactive region had a dramatic effect on the CCE curve shape at the bow near 50 V bias and a fixed space charge defined by Equation (10) would make a successful fit impossible. The corresponding fit of CCE of sample AC2 is plotted together with the experiment in Figure 14. For comparison, we also show in Figure 14 the fit with residual field neglected (*E_r_* = 0) and the obvious Hecht equation fit considering only drifting electrons and neglecting space charge. The latter case yielded a rather large *μ_e_τ_e_* = 3.9 × 10^−3^ cm^2^/V. We may see that an excellent agreement of the fit with data was reached with the presented theory. Fitted parameters were *κ* = 0.9 and the space charge density was enlarged by 9% relative to that defined in Equation (10). We simultaneously show in Figure 14 how the omission of *E_r_* affects the curve course. The deviation clearly proves the importance of *E_r_* at the CCE evaluation in case the inactive region is localized near the irradiated electrode.

## 5. Conclusions

In this paper, we compared the polarization of two p-type CdTe sensors equipped with different rectifying contacts on the anode side by the laser-induced transient technique and Am 241 gamma spectroscopy. We showed that the proper contact manufacture using Al metal can aid in the preparation of the Au/CdTe/Al sensor, revealing negligible polarization and long-term stability at a bias of up to 400 V. On the contrary, the Pt/CdTe/In detector exhibited considerable polarization incurred by the negative space charge with a density of up to 11 × 10^10^ cm^−3^ after 1 min biasing, which was varied linearly with an applied bias. In our opinion, the contact setup used by us is more convenient for the room-temperature X- and γ-ray detector applications than previous options with In- or quasi-ohmic contacts since it does not show considerable polarization whilst simultaneously preserving low leakage current. A comprehensive theoretical analysis of L-TCT and Am 241 spectroscopy data collected on both sensors allowed us to determine the drift mobility and mobility-lifetime products of both electrons and holes as well as the electric field profile in the polarizing sensor.

## Data Availability

Not applicable.
